# Identification of Immune‐Related Lactylation Genes in Rheumatoid Arthritis With Atherosclerosis: A Comprehensive Analysis Using Bulk and Single‐Cell RNA Sequencing Data

**DOI:** 10.1155/mi/9969894

**Published:** 2026-05-11

**Authors:** Jiaqi Hu, Weiyu Tao, Xinyu Qian, Qilong Liu, Zhengyi Jin, Ruina Kong, Jie Gao

**Affiliations:** ^1^ Department of Rheumatology and Immunology, Zhongshan Hospital Fudan University, Shanghai, 200032, China, fudan.edu.cn; ^2^ Department of Rheumatology and Immunology, Shanghai Changhai Hospital, The First Affiliated Hospital of Naval Medical University, Shanghai, 200433, China, smmu.edu.cn; ^3^ Department of Osteoporosis and Bone Diseases, Shanghai Jiao Tong University Affiliated Sixth People’s Hospital, Shanghai, 200433, China, sjtu.edu.cn

**Keywords:** atherosclerosis, immune infiltration, lactylation, rheumatoid arthritis

## Abstract

**Background:**

Growing evidence demonstrates that rheumatoid arthritis (RA), a chronic autoimmune disease characterized by joint inflammation and immune system dysfunction, can significantly accelerate the progression of atherosclerosis (AS). Studies have revealed that patients with RA and AS share numerous common features in terms of immune dysregulation and metabolic alterations, with abnormalities in lactate metabolism being particularly prominent. However, the role of lactate and its associated protein modification—lactylation—in the pathogenesis of RA‐related AS remains unclear. The primary objective of this study is to comprehensively investigate lactylation‐related genes as potential diagnostic markers for patients with concurrent RA and AS.

**Methods:**

We identified the core genes associated with lactylation by integrating and analyzing two disease‐related datasets: a RA dataset (GSE89408) and an AS dataset (GSE43292) from the GEO database. Through comprehensive analysis, we examined the functions associated with the hub genes and investigated the correlation between their expression levels and immune infiltration. Additionally, we explored the lactylation scores of different immune cells using single‐cell data.

**Results:**

We identified four lactylation‐related hub genes (SMARCC2, CCNA2, NUP50, and GATAD2B) highly associated with concurrent RA and AS, which showed high diagnostic potential (area under the curve [AUC] > 0.88). Further analysis revealed that these four hub genes were significantly correlated with the level of immune cell infiltration. To better understand the relationship between lactylation and immune cells, we analyzed single‐cell sequencing data, which demonstrated significant differences in lactylation scores across various types of immune cells.

**Conclusions:**

These findings highlight lactylation‐related genes as promising diagnostic markers and provide insights into shared pathogenic mechanisms of RA and AS.

## 1. Introduction

Rheumatoid arthritis (RA) is an autoimmune disease with erosive arthritis as the main clinical manifestation. The peak age of onset is 45–60 years old, and the global incidence of RA is 0.5%–1% [1]. The basic pathological changes of RA are synovitis, pannus formation, and gradual destruction of articular cartilage and bone. This eventually leads to joint deformity and loss of function [2].

Patients with RA exhibit increased incidence and mortality rates of cardiovascular diseases [3]. Atherosclerosis (AS) is characterized by the accumulation of fibrofatty lesions within arterial walls, accompanied by infiltration of immune cells (including macrophages, T cells, and mast cells), and represents the underlying cause of coronary and carotid artery diseases [4, 5]. Recent evidence suggests that RA and AS share similar pathological processes and risk factors, with chronic inflammation and immune dysfunction being the most prominent [6, 7]. Although the underlying mechanisms linking RA and AS remain unclear, it is evident that both conditions involve chronic inflammation and immune infiltration. AS is an inflammatory process that can lead to plaque rupture, thrombosis, and vascular occlusion [8]. In RA patients, immune processes may begin years before diagnosis, during the pre‐RA phase [9]. Moreover, many pathological processes in the arterial walls during AS are mirrored in RA synovial inflammation, including the infiltration of macrophages and type 1 helper T cells, which secondarily affect arteries through mediators produced in the synovium [7]. Therefore, identifying immune infiltration patterns and associated inflammatory molecules may have early diagnostic value for RA patients with concurrent AS, which is crucial for preventing severe cardiovascular consequences.

Immune system dysregulation is considered one of the most critical factors in the pathogenesis of RA, with recent research suggesting that alterations in immune cell metabolic pathways may play a more significant role in RA progression than previously recognized [10]. Furthermore, disrupted organic acid metabolism has been a longstanding area of investigation in RA patients, with studies documenting notably elevated lactate concentrations in the synovial environment.

Historically, lactate was long viewed as merely a metabolic waste product of glycolysis [11, 12]. However, in 2019, Zhao’s research demonstrated that lactate accumulated during metabolism can serve as a precursor for histone lysine lactylation modifications, which regulate gene expression and participate in maintaining the homeostasis of M1 macrophages during bacterial infection [13]. In 2022, the research team led by Hao Haiping and Ye Hui made significant advances by discovering novel lactylation substrate proteins and modification sites. Unlike other metabolic modifications, lactylation directly links lactate accumulation (a hallmark of RA synovial inflammation [14]) to epigenetic and transcriptional regulation of immune cells [13, 15], making it a critical but understudied target in RA‐related AS. However, research on lactate‐related gene expression and lactate modification in RA‐related AS is still in its infancy, with no relevant studies reported to date. In the context of RA chronic inflammation, lactate accumulation in the tissue microenvironment induces human CD4+ T cells to upregulate the lactate transporter SLC5A12, leading to effector phenotype reprogramming and increased IL‐17 production, causing CD4+ T cells to be retained within the inflamed tissue and exacerbating tissue inflammation [16]. In contrast to its significant inflammatory‐inducing and promoting effects in RA, lactate exhibits protective effects in mouse models of autoimmune hepatitis and pancreatitis [17]. In vivo experiments have demonstrated that high levels of lactate can modulate both the metabolic and epigenetic states of Th17 cells, significantly reducing their IL‐17A production while upregulating the Foxp3 expression. Lactate can effectively reprogram proinflammatory T cells into regulatory T cells (Tregs) [18]. Recent studies have shown that lactylation is also closely associated with other autoimmune diseases, including systemic lupus erythematosus and ulcerative colitis [19, 20]. However, no studies have systematically identified lactylation‐related genes or their roles in immune infiltration across both RA and AS, leaving a gap in the diagnostic and therapeutic strategies for comorbid patients.

In this study, we employed a comprehensive analytical approach integrating bulk RNA sequencing (RNA‐seq) and single‐cell RNA‐seq (scRNA‐seq) data to identify key lactylation‐associated hub genes in patients with comorbid RA and AS. Furthermore, we explored the intricate relationship between the immune microenvironment (IME) and lactylation patterns in this disease intersection. This research aims to unveil novel potential biomarkers for patients with concurrent RA and AS, providing valuable insights into the shared pathogenic mechanisms and potentially opening new therapeutic avenues for this specific patient population.

## 2. Methods

### 2.1. Identification of Differences in Expression Genes Between RA and AS Patients

Two disease‐relevant gene expression matrices (GSE43292 for AS and GSE89408 for RA) were obtained from the GEO database (https://www.ncbi.nlm.nih.gov/geo/). For GSE43292, we analyzed 32 atherosclerotic plaque samples and 32 matched control arterial tissues. For GSE89408, which contains synovial biopsies from multiple arthritis conditions, we selected 152 RA samples and 28 healthy controls and excluded samples from OA (*n* = 22), arthralgia (*n* = 10), and undifferentiated arthritis (*n* = 6).

Because each disease was analyzed using a single independent dataset and no dataset merging was performed, no cross‐dataset batch effect correction was required. Each dataset was independently processed following the standard GEO analysis workflow, including background correction, log_2_ transformation, and quantile normalization. Differentially expressed genes (DEGs) were identified using linear models with empirical Bayes moderation implemented in the limma package. This approach is particularly suitable for datasets with modest sample sizes, such as GSE43292, as it stabilizes variance estimates across genes. *p*‐Values were adjusted for multiple testing using the Benjamini–Hochberg false discovery rate (FDR).

### 2.2. The Expression Feature of Lactylation‑Related Genes

Three hundred thirty‐six lactylation‐related genes were identified through Gene Ontology (GO) (http://geneontology.org/) and previous literature (PMID: 37242427 35761067 36092712). Among these, 44 genes showing significant differential expression between both RA and AS patients compared to healthy controls were selected for further analysis. Protein–protein interaction (PPI) networks for these 44 genes were constructed using the STRING database. To identify the most relevant genes, we employed a multialgorithmic approach. First, we utilized the least absolute shrinkage and selection operator (LASSO) regression, implemented through the “glmNETs” R package. LASSO, a sophisticated form of multivariate linear regression, incorporates a penalty function to prevent overfitting and manage covariance, resulting in the selection of 11 key genes. Subsequently, we applied random forest classification and XGBoost analysis using the “randomForest” and “XGBoost” R packages, respectively, retaining the top 15 genes from each method. The final set of lactylation‐related hub genes was determined by identifying the intersection of results from LASSO regression, random forest, and XGBoost analyses. The diagnostic potential of these hub genes in both RA and AS was evaluated using receiver operator characteristic (ROC) curves, with area under the curve (AUC) values calculated using the R package “pROC.” To understand the biological significance of these findings, we performed functional enrichment analyses, including GO and Kyoto Encyclopedia of Genes and Genomes (KEGG) pathway analyses, using the R package “clusterProfiler.”

### 2.3. Association Between Lactylation Hub Genes and Immunological Characteristics

To comprehensively evaluate immune cell infiltration patterns across samples, we employed the ssGSEA algorithm implemented through the R package “GSVA.” Subsequently, we conducted Spearman correlation analyses to investigate the relationships between hub genes and infiltrating immune cells. Unsupervised consensus clustering of RA samples based on the expression of the four hub genes was performed using the R package ConsensusClusterPlus to identify potential molecular subtypes.

### 2.4. Gene Set Enrichment Analysis (GSEA) and Regulatory Network Construction

We first investigated the coexpression patterns among hub genes using Spearman correlation analysis. GSEA was performed on the Hallmark gene sets (http://software.broadinstitute.org/gsea/msigdb/) using the R package “clusterProfiler,” focusing on significantly coexpressed genes (*p* < 0.05). To elucidate the regulatory mechanisms, we utilized the regnetwork database to identify upstream miRNAs and transcription factors and subsequently constructed comprehensive regulatory networks using Cytoscape software.

### 2.5. Consensus Clustering Analysis

Unsupervised consensus clustering of RA samples based on the expression of the four hub genes was performed using the R package ConsensusClusterPlus to identify potential molecular subtypes.

### 2.6. scRNA‑seq Analysis

scRNA‐seq data were obtained from a previously published dataset, which included synovial immune cells from 20 RA patients and five healthy controls, covering major immune cell populations such as T cells, monocytes, and B cells [21]. Given the multisample nature of the scRNA‐seq dataset and the presence of donor‐specific technical variability, batch effects across samples were mitigated using the R package Harmony. Data normalization and scaling were performed using the ScaleData function.

Principal component analysis (PCA) was conducted on the normalized data, followed by dimensionality reduction using RunUMAP. Differential gene expression across cell clusters was identified using the FindAllMarkers function, and cell type annotation was performed with the SingleR package. To evaluate lactylation‐associated patterns, the GSVA package was used to calculate lactylation enrichment scores at the single‐cell level (derived from GSVA‐based lactylation gene sets). In addition, cell type–specific pathway enrichment scores were computed based on the Hallmark gene set database. Correlation analyses were subsequently performed between pathway enrichment scores and lactylation scores. To further explore biological processes, pathway enrichment analyses were conducted using the KEGG and Reactome databases, focusing on the top 100 marker genes identified for each cell population.

### 2.7. Animals

A total of 12 8‐week‐old male DBA/1 mice were used in this study, with six mice in the collagen‐induced arthritis (CIA) group and six mice in the control group. The CIA model was established following previously described protocols. Mice in the CIA group received a primary immunization via tail‐root injection with 100 μL of an emulsion containing bovine type II collagen (10 mg, Chondrex, #20021) and complete Freund’s adjuvant (4 mg/mL, Chondrex, #7001) at a 1:1 ratio, followed by a booster immunization on day 21 using collagen emulsified with incomplete Freund’s adjuvant (Chondrex, #7002). Arthritis progression was monitored every 2 days. After a 21‐day observation period, mice were euthanized, and synovial tissues were collected, rinsed in cold PBS, snap‐frozen in liquid nitrogen, and stored at −80°C for further analysis. All animal experimental procedures were reviewed and approved by the Animal Care and Use Committee of Shanghai Changhai Hospital (Approval Number CHEC2020‐105) and were conducted in accordance with the National Institutes of Health Guide for the Care and Use of Laboratory Animals.

### 2.8. RNA Isolation and Quantitative Real‐Time PCR (qRT‐PCR)

Total RNA was extracted from the samples using the TRIzol reagent. Using HiScript II Q RT SuperMix (Vazyme, China, #R222‐02), 1 µg of RNA was reverse transcribed into cDNA. qRT‐PCR was performed on an Applied Biosystems LightCycler‐480 instrument (Roche, USA) using SYBR qPCR Master Mix (Sangon Biotech, China) with specific primers. All primer sequences used in this study are detailed in Supporting Information 1: Table S1.

### 2.9. Western Blot

Primary antibodies were incubated with the membranes overnight at 4°C, including GATAD2B (1:1000, Proteintech, 25679‐1‐AP), SMARCC2 (1:1000, Proteintech, 12018‐1‐AP), NUP50 (1:1000, Proteintech, 20798‐1‐AP), CCNA2 (1:1000, Proteintech, 18202‐1‐AP), and GAPDH (1:1000, Abcam, ab16891). The membranes were then incubated with an HRP‐conjugated antirabbit secondary antibody (CST 7074, 1:5000) for signal detection. Protein bands were visualized using a Tanon 5200Mui.

### 2.10. Statistical Analysis

All experimental data are presented as mean ± standard deviation (SD). GraphPad Prism (version 9.0) was used for general statistical analyses, while differential expression analyses were conducted using R software (version 4.4.1). Comparisons between two groups were conducted using an unpaired two‐tailed Student’s *t*‐test. Comparisons among multiple groups were analyzed by one‐way ANOVA, followed by Tukey’s post hoc test for pairwise comparisons. A two‐tailed *p*‐value < 0.05 was considered statistically significant. For differential expression analyses, effect sizes (e.g., log_2_ fold change), 95% confidence intervals, and adjusted *p*‐values (FDR) were reported.

## 3. Results

### 3.1. Differential Gene Expression Analysis Reveals Distinct Molecular Signatures in RA and AS Patients

**Figure 1:**
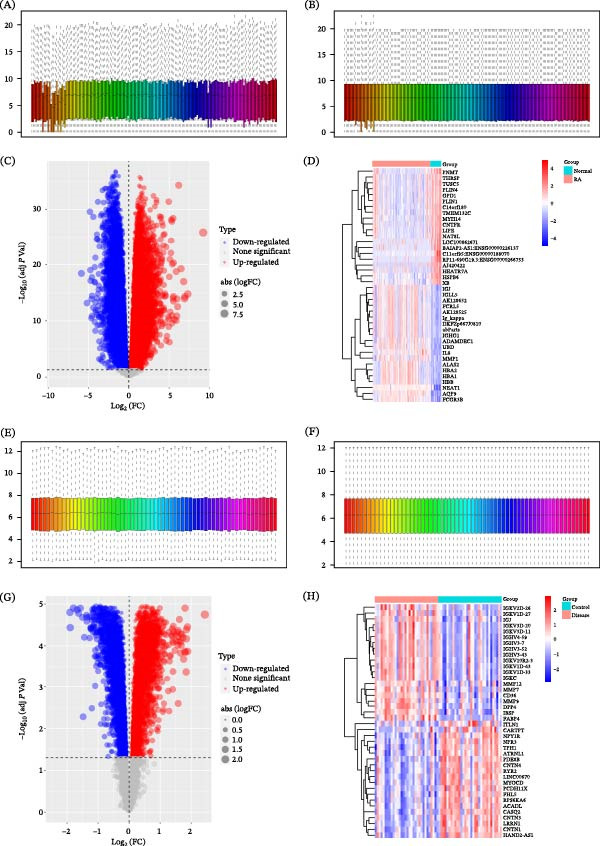
Differential gene expression analysis in RA and AS. (A, B) Visualization of the RA dataset (GSE89408) before (A) and after (B) normalization using the R preprocessCore package. (C) Volcano plot of DEGs in RA. Significantly upregulated (adj.*p*.Val < 0.05, log_2_FC > 0) and downregulated (adj.*p*.Val < 0.05 and log_2_FC < 0) genes are highlighted in red and blue, respectively. (D) Heatmap of the top 20 DEGs distinguishing RA patients from healthy controls. (E, F) Visualization of the AS dataset (GSE43292) before (E) and after (F) normalization. (G) Volcano plot of DEGs in AS, with significant genes colored as in (C). (H) Heatmap of the top 20 DEGs distinguishing AS patients from healthy controls.

After obtaining the RA‐related dataset (GSE89408), we performed standard preprocessing and first visualized the data quality. The effect of normalization on the dataset is shown, illustrating the removal of outlier effects (Figure 1A,B). Subsequently, gene expression profiles were analyzed for 152 RA patients and 28 healthy controls. A total of 17,291 DEGs were identified at an FDR‐adjusted *p*  < 0.05, including 9085 upregulated and 8206 downregulated genes (Figure 1C). Heatmap visualization demonstrated a clear separation between RA patients and healthy controls based on the expression patterns of the top DEGs (Figure 1D). Similarly, the AS‐related dataset (GSE43292) was processed using the same workflow. Data normalization was applied and visualized (Figure 1E,F), yielding expression profiles from 32 AS patients and 32 healthy controls. In the AS dataset, 6730 DEGs were identified at an FDR‐adjusted *p*  < 0.05, including 3220 upregulated and 3510 downregulated genes (Figure 1G). The AS‐specific transcriptional signature based on the top DEGs effectively distinguished disease samples from controls (Figure 1H), with representative genes such as immunoglobulin variable genes (IGKVs) and matrix metalloproteinases (MMPs) showing distinct expression patterns between groups.

### 3.2. Overlap Analysis Reveals Shared Lactylation‐Associated Genes and Pathways Between RA and AS

**Figure 2:**
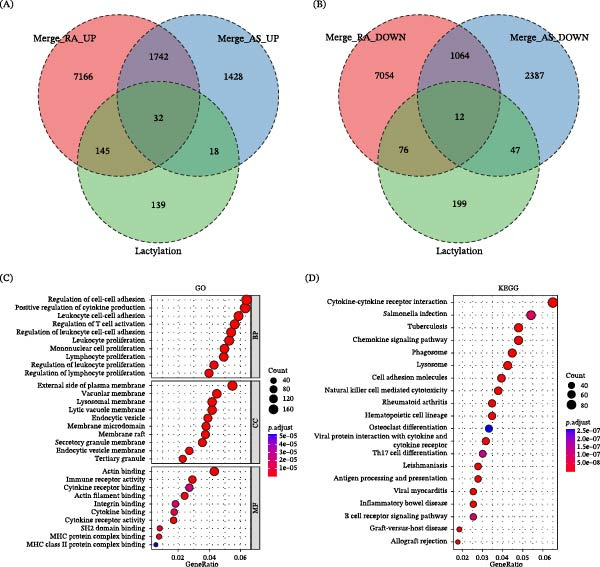
Overlap analysis of lactylation‐associated differentially expressed genes in RA and AS. (A, B) Venn diagrams showing the overlap of upregulated (A) and downregulated (B) genes in RA and AS with a curated lactylation‐associated gene set. (C) GO enrichment analysis of genes commonly dysregulated in both RA and AS. (D) KEGG pathway enrichment analysis of genes commonly dysregulated in both RA and AS.

Given the distinct molecular signatures observed in RA and AS, we next sought to identify common pathological mechanisms that might underlie both diseases. We therefore focused on lactylation, a recently recognized posttranslational modification linked to inflammation and metabolism. To this end, we performed overlap analyses between disease‐related DEGs and a curated lactylation‐associated gene set. Distinct overlap patterns were observed for both upregulated and downregulated genes. Among upregulated genes, 1742 genes were shared between RA and AS, of which 32 genes overlapped with the lactylation‐associated gene set (Figure 2A). Among downregulated genes, 1064 genes were common to both diseases, with 12 genes intersecting with lactylation‐associated genes (Figure 2B). To investigate the biological relevance of the shared disease‐related genes, GO enrichment analysis was performed on the overlapping RA–AS gene set. The results revealed significant enrichment in immune‐related biological processes, including regulation of cell–cell adhesion, positive regulation of cytokine production, leukocyte cell–cell adhesion, and regulation of T cell activation (Figure 2C). Consistently, KEGG pathway analysis demonstrated enrichment in key immune and inflammatory pathways, such as cytokine–cytokine receptor interaction, chemokine signaling pathway, and tuberculosis, with particularly prominent enrichment in RA‐related pathways (Figure 2D).

### 3.3. Functional Enrichment Analysis of Shared Lactylation Genes Reveals Metabolic and Immune Pathways

**Figure 3:**
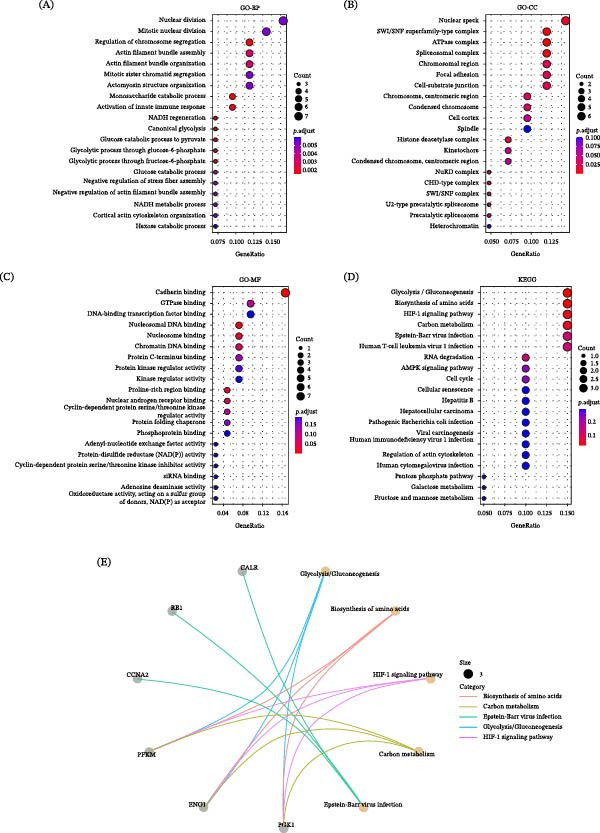
GO and KEGG enrichment analysis of the 44 shared genes among RA, AS, and lactylation. (A) GO enrichment analysis of biological processes. (B) GO enrichment analysis of cellular components. (C) GO enrichment analysis of molecular functions. (D) KEGG pathway enrichment analysis. (E) Network showing the correspondence between the top 5 enriched KEGG pathways and their associated genes.

We performed comprehensive functional analysis of the 44 overlapping genes identified across diseases and lactylation. GO biological process analysis revealed significant enrichment in nuclear division, mitotic nuclear division, and regulation of chromosome segregation pathways (Figure 3A). Cellular component analysis highlighted enrichment in nuclear structures, including nuclear speck and SWI/SNF superfamily–type complex, as well as cytoskeletal components (Figure 3B). Molecular function analysis showed enrichment in cadherin binding and various enzymatic activities (Figure 3C). KEGG pathway analysis identified significant enrichment in metabolic pathways, particularly glycolysis/gluconeogenesis, biosynthesis of amino acid, and HIF‐1 signaling (Figure 3D). To further elucidate the specific gene constituents of these key pathways, we employed the STRING database to forecast protein interactions and constructed a network diagram illustrating the associations between the top 5 enriched KEGG pathways and their corresponding genes from the overlapping set (Figure 3E).

### 3.4. Identification of Hub Genes of Lactylation

**Figure 4:**
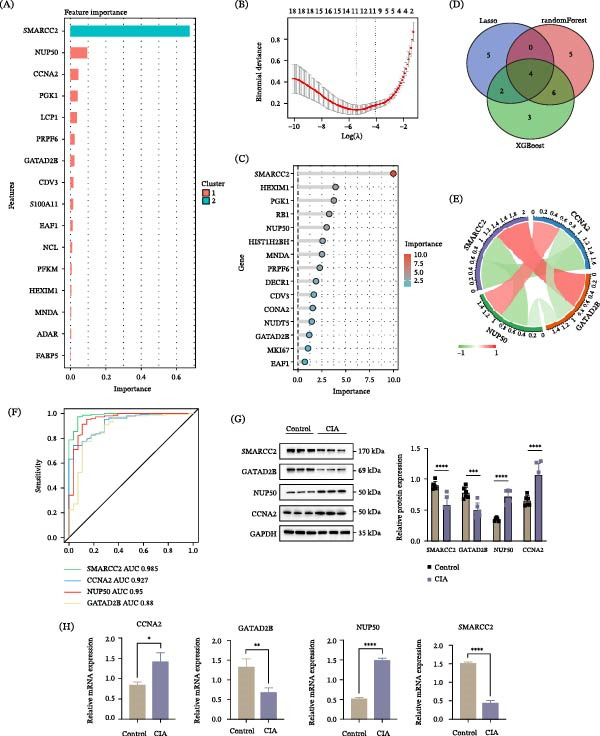
Identification of four lactylation‐associated hub genes in RA. (A) Feature importance ranking generated by the XGBoost algorithm. (B) LASSO regression analysis identifying 11 candidate genes at the optimal lambda value. (C) Random forest–based importance ranking of candidate genes. (D) Venn diagram showing the intersection of genes identified by XGBoost, LASSO regression, and random forest analysis, yielding four hub genes. (E) Correlation analysis among the four hub genes, where red indicates positive correlation and green indicates negative correlation. (F) ROC curves evaluating the diagnostic performance of the four hub genes for RA. (G–H) The protein expression (G) and mRNA expression (H) of four hub genes in synovial tissues of CIA and normal knees(*n* = 6 per group). Data are presented as mean ± SEM. Significance levels are indicated as follows: ns (not significant),  ^∗^ *p* < 0.05,  ^∗∗^ *p* < 0.01,  ^∗∗∗^ *p* < 0.001, and  ^∗∗∗∗^ *p* < 0.0001.

To identify key lactylation‐associated hub genes in RA, multiple machine learning approaches were applied to the 44 overlapping genes identified from the intersection analysis. Using the XGBoost algorithm, SMARCC2 was ranked as the most important feature based on feature importance scores (Figure 4A). LASSO regression analysis selected 11 candidate genes at the optimal lambda value (Figure 4B). In parallel, a random forest model ranked genes according to their relative importance, with SMARCC2 again showing the highest importance score (Figure 4C). To enhance robustness, genes consistently identified across different algorithms were prioritized. The intersection of results from the three methods—specifically, the LASSO‐selected genes, the top 15 genes from random forest, and the key features from XGBoost—yielded four hub genes: SMARCC2, CCNA2, NUP50, and GATAD2B (Figure 4D). Correlation analysis revealed significant associations among these four hub genes (Figure 4E). To explore their potential diagnostic relevance in RA, ROC curve analysis was performed. SMARCC2 demonstrated the highest predictive performance (AUC = 0.985), followed by CCNA2 (AUC = 0.927), NUP50 (AUC = 0.950), and GATAD2B (AUC = 0.880) (Figure 4F). Notably, these ROC analyses were conducted in the same dataset and therefore reflect exploratory diagnostic potential rather than independent validation. To further support the bioinformatics findings, a CIA mouse model was established, and Western blot and qPCR analysis of synovial tissues confirmed that the expression patterns of these four hub genes were consistent with the computational results (Figure 4G,H).

### 3.5. Functional Analysis of Hub Genes in RA Patients

**Figure 5:**
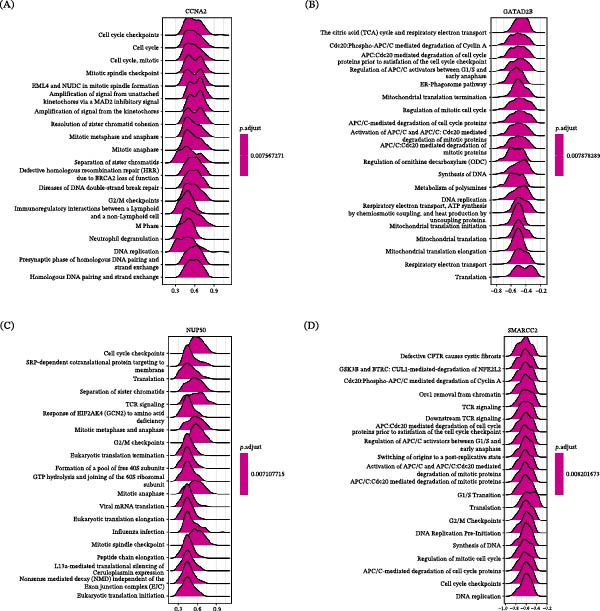
Correlation‐based single‐gene GSEA of hub genes in RA.GSEA based on gene expression correlation rankings for (A) CCNA2, (B) GATAD2B, (C) NUP50, and (D) SMARCC2 using the Reactome pathway database. The top 20 enriched pathways are shown. Positive enrichment scores indicate positive correlation with the hub gene, whereas negative enrichment scores indicate negative correlation.

To further explore the biological relevance of the identified hub genes in RA, we performed genome‐wide correlation analyses for each gene. Based on the resulting correlation‐ranked gene lists, we conducted single‑gene GSEA against the Reactome pathway database (Supporting Information 2: Additional file S2). CCNA2 was positively associated with pathways related to cell cycle regulation, DNA replication, and mitotic progression, with notable enrichment in the resolution of sister chromatid cohesion and mitotic spindle checkpoint pathways (Figure 5A). In contrast, GATAD2B showed predominantly negative associations with mitochondrial function and energy metabolism, including the tricarboxylic acid (TCA) cycle, respiratory electron transport chain, and APC/C‐mediated protein degradation pathways (Figure 5B). NUP50 exhibited positive enrichment patterns in pathways involved in protein translation and cell cycle checkpoints, including mitotic metaphase and anaphase and viral mRNA translation pathways (Figure 5C). Conversely, SMARCC2 was negatively associated with pathways related to cell cycle control, chromatin organization, and immune signaling, including DNA replication, T cell receptor signaling, and cell cycle checkpoint pathways (Figure 5D).

### 3.6. The Expression of Hub Genes Correlated With Immunological Characteristics in RA Patients

**Figure 6:**
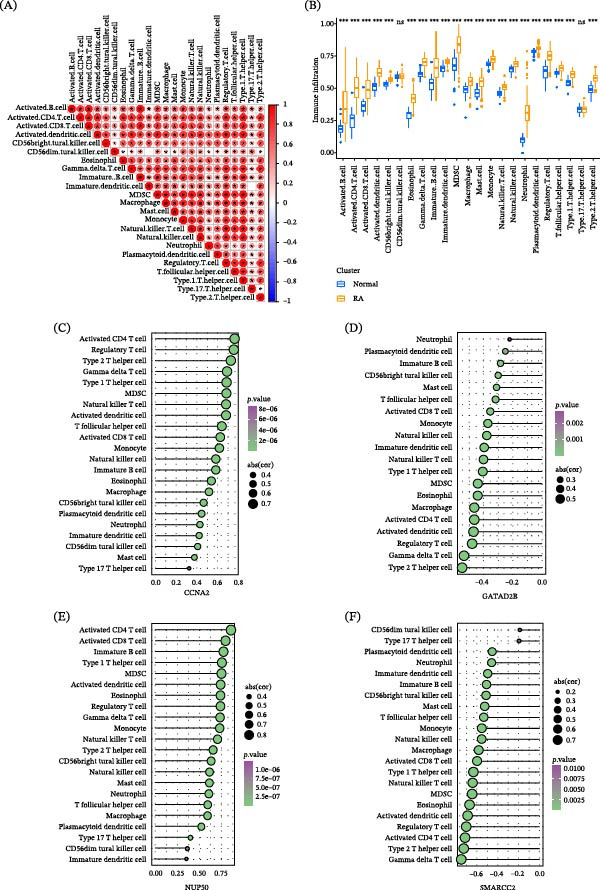
Association between hub gene expression and immune cell infiltration in RA. (A) Correlation matrix of immune cell infiltration scores estimated by ssGSEA in RA samples. (B) Comparison of immune cell infiltration levels between RA patients and healthy controls. (C–F) Correlation between immune cell infiltration scores and the expression levels of (C) CCNA2, (D) GATAD2B, (E) NUP50, and (F) SMARCC2. Only immune cell types with statistically significant correlations are displayed. Circle size represents the correlation coefficient, and color indicates the *p*‐value. Data are presented as mean ± SEM. Significance levels are indicated as follows: ns (not significant),  ^∗^ *p* < 0.05,  ^∗∗^ *p* < 0.01,  ^∗∗∗^ *p* < 0.001, and  ^∗∗∗∗^ *p* < 0.0001.

To investigate the immunological characteristics of RA, immune cell infiltration levels were estimated using the ssGSEA method. Correlation analysis revealed extensive associations among different immune cell populations in RA samples, with positive correlations predominating (Figure 6A). Comparative analysis demonstrated that the infiltration levels of the majority of immune cell types were significantly increased in RA patients compared with healthy controls, whereas no immune cell type showed a statistically significant decrease (Figure 6B). Further correlation analyses were performed between the expression levels of the four hub genes and immune cell infiltration scores. CCNA2 expression was positively correlated with the infiltration levels of most immune cell populations (Figure 6C). In contrast, GATAD2B expression showed predominantly negative correlations with immune cell infiltration (Figure 6D). NUP50 expression was also positively associated with immune cell infiltration across multiple immune cell types (Figure 6E), whereas SMARCC2 expression exhibited predominantly negative correlations with immune cell infiltration levels (Figure 6F).

### 3.7. Consensus Clustering Reveals Two Molecular Subtypes in RA With Distinct Pathway Activity Patterns

**Figure 7:**
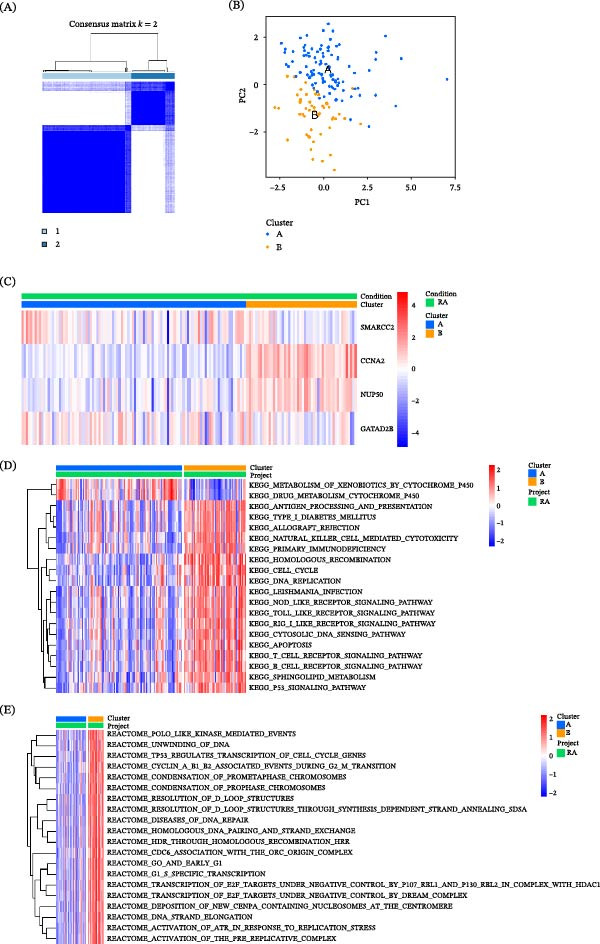
Consensus clustering and pathway activity analysis based on four hub genes in RA. (A) Consensus clustering matrix showing classification of RA samples into two clusters using ConsensusClusterPlus. (B) PCA plot illustrating the distribution of samples in the two clusters. (C) Heatmap showing the expression patterns of the four hub genes across the two clusters. (D) GSVA‐based comparison of KEGG pathway activity between the two clusters. (E) GSVA‐based comparison of Reactome pathway activity between the two clusters.

Based on the expression profiles of the four hub genes, we performed unsupervised consensus clustering using the R package ConsensusClusterPlus to explore potential molecular subtypes in RA. The consensus clustering results indicated that classification into two clusters was optimal (Figure 7A).

PCA showed an apparent separation between the two clusters in the reduced dimensional space, supporting the robustness of the clustering results (Figure 7B). The expression patterns of the four hub genes differed markedly between Cluster A and Cluster B, as illustrated by the heatmap (Figure 7C). To further characterize the biological differences between the two clusters, we conducted GSVA using KEGG and Reactome pathway gene sets. KEGG pathway analysis revealed distinct pathway activity patterns between the two clusters, with one cluster showing relatively higher enrichment in immune‐related pathways, including T cell receptor signaling, natural killer (NK) cell–mediated cytotoxicity, and primary immunodeficiency, while metabolic pathways such as xenobiotic metabolism and cytochrome P450 also displayed differential enrichment (Figure 7D). Reactome pathway analysis similarly demonstrated widespread differences in pathway activity between the two clusters (Figure 7E). In addition, to explore potential upstream regulatory relationships of the hub genes, we constructed a gene regulatory network based on the RegNetwork database, incorporating transcription factors and miRNAs. The resulting network highlighted complex regulatory interactions involving GATAD2B, SMARCC2, and NUP50 (Supporting Information 3: Additional file S3).

### 3.8. scRNA‐seq Analysis Reveals Transcriptionally Distinct Immune Cell Populations in RA

**Figure 8:**
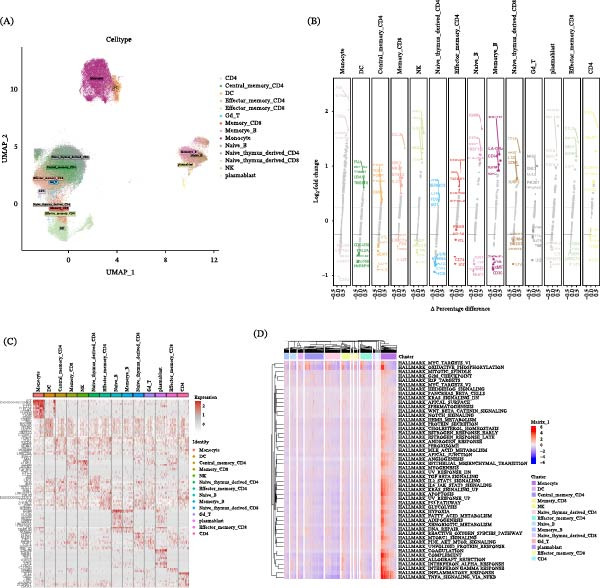
Single‐cell RNA‐seq analysis of immune cell populations in RA. (A) Cell type annotation results based on Seurat clustering. (B) Top five differentially expressed genes identified by FindAllMarkers for each cell type. (C) Heatmap showing the top 10 marker genes for each cell type identified by COSG. (D) Heatmap of Hallmark pathway GSVA scores across different immune cell populations.

scRNA‐seq data were processed and analyzed using the Seurat pipeline. After stringent quality control, normalization, and dimensionality reduction, cells were clustered into 14 transcriptionally distinct populations (Figure 8A). These clusters were annotated as specific immune cell types and subsets based on the expression of canonical marker genes. The identified populations included CD4+ T cells (naive, central memory, and effector memory), CD8+ T cells (naive and memory), B cells (naive and memory), plasmablasts, NK cells, dendritic cells (DCs), monocytes, and γδT cells. To define the identity of each cluster, we performed differential expression analysis using the FindAllMarkers function. The top five upregulated marker genes for each cluster are displayed in a volcano plot (Figure 8B). For a more stringent identification of cell type–specific signatures, we applied the COSG algorithm. A heatmap of the top 10 marker genes identified by COSG clearly illustrates the distinct expression profiles across clusters (Figure 8C). Furthermore, functional enrichment analyses of the top 100 marker genes from each cell cluster were conducted using the R package clusterProfiler. The enriched KEGG and Reactome pathways are presented in Supporting Information 4: Additional file S4.To investigate the functional heterogeneity among these immune cell populations, we performed GSVA using the Hallmark gene sets from the MSigDB database. The resulting GSVA score heatmap (Figure 8D) reveals distinct pathway activity patterns across clusters. For instance, monocyte populations showed enrichment in inflammatory pathways such as TNFA_SIGNALING_VIA_NFKB and INFLAMMATORY_RESPONSE, whereas T cell clusters exhibited elevated activity in IL2_STAT5_SIGNALING and T cell receptor–related pathways.

### 3.9. Single‐Cell Analysis Reveals Cell Type–Specific Lactylation Patterns and Associated Pathways in RA

**Figure 9:**
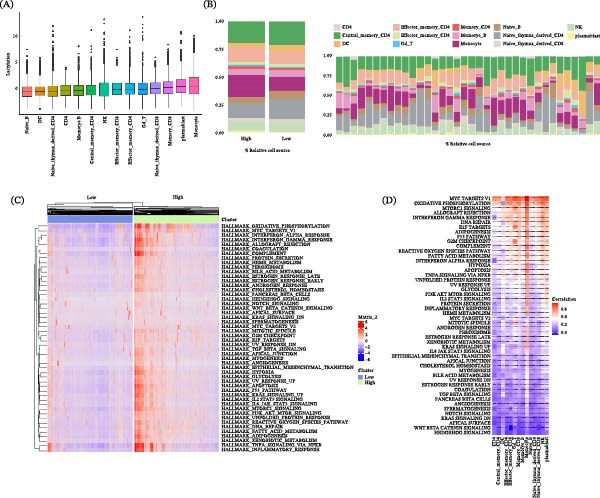
Single‐cell lactylation profiling and pathway association analysis in RA. (A) GSVA‐based lactylation gene set scores across different immune cell types. (B) Cellular composition and sample‐wise distribution of high‐ and low‐lactylation groups. (C) Heatmap showing Hallmark pathway GSVA scores comparing high‐ and low‐lactylation cells. (D) Correlation heatmap between lactylation scores and Hallmark pathway enrichment scores.

To characterize lactylation at the single‐cell level, we calculated lactylation scores for individual cells using GSVA based on a curated set of 336 lactylation‐related genes. This analysis revealed distinct lactylation scores across immune cell types (Figure 9A). Central memory CD4+ T cells, naïve thymus–derived CD4+ T cells, and naïve B cells exhibited the lowest scores, whereas monocytes, effector memory CD4+ T cells, and CD8+ T cell subsets showed the highest lactylation scores (Figure 9A). To determine whether lactylation‐high cells are enriched in specific cellular compartments, we compared the proportion of each cell type between cells stratified into high‐ and low‐lactylation groups based on the median lactylation score. The high‐lactylation group was markedly enriched for monocytes and effector T cells (Figure 9B, left panel). The sample‐wise distribution of these cellular proportions is also shown (Figure 9B, right panel). To identify the biological states associated with high lactylation, we compared Hallmark pathway activities (assessed by GSVA) between the high‐ and low‐lactylation groups. The high‐lactylation group showed significant upregulation of pathways including OXIDATIVE_PHOSPHORYLATION, INFLAMMATORY_RESPONSE, and TNFA_SIGNALING_VIA_NFKB (Figure 9C). Finally, to systematically pinpoint the pathways most tightly linked to lactylation, we calculated the correlation between the lactylation score and each Hallmark pathway score across all single cells. The pathways exhibiting the strongest positive correlations were OXIDATIVE_PHOSPHORYLATION, MYC_TARGETS_V1, and MTORC1_SIGNALING (Figure 9D). Together, these data establish a strong association between elevated protein lactylation and a coordinated upregulation of immunometabolic and inflammatory pathways in key pathogenic immune cells within the RA synovium.

## 4. Discussion

RA is a heterogeneous autoimmune disease characterized by persistent synovial inflammation and aberrant immune activation [22]. Accumulating evidence suggests that metabolic reprogramming plays a critical role in shaping immune responses during RA pathogenesis [23]. While previous studies have largely focused on dietary factors and microbial metabolites [24–27], the contribution of endogenous metabolites—particularly lactate and lactylation‐related modifications—to immune dysregulation remains incompletely understood [13, 28]. In this study, we systematically investigated lactylation‐associated genes in RA and AS by integrating bulk RNA‐seq and scRNA‐seq analyses with experimental validation and revealed that lactylation‐associated transcriptional signatures are closely associated with aberrant immune activation across key immune cell populations.

Through differential expression and overlap analyses, we identified four lactylation‐associated hub genes—SMARCC2, CCNA2, NUP50, and GATAD2B—that were consistently dysregulated in both RA and AS and closely correlated with immune infiltration patterns. Machine learning approaches further supported the robustness of these genes as potential diagnostic markers, highlighting their relevance to shared immunometabolic mechanisms underlying RA–AS comorbidity. Currently, the diagnosis of RA requires a systematic evaluation of clinical, laboratory, and radiological findings [13], and early diagnosis significantly reduces the risk of adverse clinical outcomes [28]. Our findings suggest that these hub genes show potential as candidate biomarkers for distinguishing RA patients from healthy individuals.

Among the identified hub genes, CCNA2 is a key regulator of cell cycle progression and has been implicated in the abnormal proliferation and apoptosis resistance of fibroblast‐like synoviocytes (FLS) in RA, contributing to joint destruction [29]. Dysregulated CCNA2 expression may indirectly sustain synovial inflammation by influencing immune cell proliferation, survival, and the expression of MMPs through interactions with signaling networks such as NF‐κB and MAPK pathways [30–33]. SMARCC2 and GATAD2B, both involved in chromatin remodeling, are likely to affect inflammatory gene expression programs through epigenetic mechanisms. Our analyses revealed decreased SMARCC2 expression in RA, which may impair the suppression of immune cell activation and enhance proinflammatory cytokine production (e.g., TNF‐α and IL‐6) via pathways such as NF‐κB [34–36]. Conversely, NUP50 upregulation may contribute to immune activation by modulating nucleocytoplasmic transport and cytokine signaling. NUP50 expression is positively correlated with T cell and B cell activity, potentially leading to increased autoantibody production and proinflammatory cytokine secretion (e.g., TNF‐α, IL‐6, and IL‐17) while negatively correlated with regulatory T cell function [37–41]. GATAD2B downregulation is associated with the pathogenic phenotype of RA FLS and the activation of proinflammatory M1 macrophages while also correlating with the expansion of Tregs [42–45]. Collectively, these findings suggest that lactylation‐related genes preferentially converge on nuclear and chromatin‐associated processes to shape inflammatory transcriptional programs. This observation is consistent with recent proteomic evidence demonstrating that lactylation preferentially targets nuclear and chromatin‐associated proteins to regulate inflammatory transcriptional programs [46]. Our findings extend this work by showing that transcriptional dysregulation of chromatin‐remodeling genes associated with lactylation is detectable at both bulk and single‐cell levels in RA and AS.

scRNA‐seq analysis further revealed pronounced cell type–specific heterogeneity in lactylation scores. Monocytes, effector memory CD4+ T cells, memory CD8+ T cells, and naive thymus–derived CD8+ T cells exhibited relatively higher lactylation scores. These immune cell subsets are well recognized as major drivers of synovial inflammation, supporting a close association between the lactylation status and immune activation in RA [7]. Pathway analyses demonstrated that lactylation scores were strongly correlated with oxidative phosphorylation, MYC targets, and mTORC1 signaling [47, 48], consistent with emerging evidence that lactylation acts as a key immunometabolic regulator linking cellular metabolism to inflammatory gene expression. KEGG analysis also indicated a correlation with pyruvate metabolism, tight junction, and apoptotic pathways [49, 50], which are known to play crucial roles in synovial homeostasis and RA pathogenesis.

Lactylation has emerged as a critical immunometabolic modification that directly links cellular metabolic states to immune gene regulation. Unlike traditional metabolic byproducts, lactate‐derived lactylation actively reshapes chromatin accessibility and transcriptional programs in immune cells, thereby modulating inflammatory responses [51]. In inflammatory settings, elevated glycolytic flux and lactate accumulation promote lactylation‐driven activation of proinflammatory gene networks, particularly in myeloid cells and effector T cells. Our findings support this concept by demonstrating that higher lactylation scores are associated with metabolic pathways such as oxidative phosphorylation and MYC signaling, highlighting lactylation as an active driver—rather than a bystander—of immune dysregulation in RA and AS.

Recent studies have highlighted lactylation as an important epigenetic modification that modulates immune cell function by reshaping chromatin accessibility and transcriptional activity [50, 51]. Building on these findings, our results demonstrate that lactylation‐associated genes are not only dysregulated in RA but are also shared with AS, suggesting a convergent immunometabolic mechanism underlying chronic inflammatory diseases. Notably, RA and AS share overlapping inflammatory pathways, including TNF‐α signaling, Th17‐mediated immune responses, and metabolic reprogramming of innate immune cells. The identification of common lactylation‐associated hub genes suggests that lactylation may act as a convergent immunometabolic mechanism underlying these shared inflammatory features. This suggests that lactylation‐related immunometabolic mechanisms may represent a common pathogenic link between chronic inflammatory diseases rather than disease‐specific phenomena. Compared with previous studies focused on single‐disease contexts or specific cell types, our work provides a systemic, multiomics perspective on lactylation in RA–AS comorbidity.

Several limitations of this study should be acknowledged. First, the relatively small sample size of the AS dataset (GSE43292) may limit the detection of genes with modest effect sizes [52, 53]. Nevertheless, the use of empirical Bayes modeling within the limma framework helps stabilize inference in smaller datasets [54, 55], and the identified signatures are consistent with prior studies of similar scales, supporting their reliability. Second, while our results are supported by animal validation at the transcript and protein levels, and our findings indicate that lactylation‐related genes may serve as potential diagnostic markers for RA–AS comorbidity, further mechanistic studies and validation in larger independent clinical cohorts are required to confirm their biological and diagnostic relevance.

In summary, our integrative multiomics analysis identifies lactylation‐associated genes as potential shared regulators of immune dysregulation in RA and AS. These findings provide new insights into the immunometabolic mechanisms underlying chronic inflammatory diseases and offer a foundation for future studies targeting lactylation‐related pathways for therapeutic intervention.

## Author Contributions

**Jiaqi Hu**: writing – original draft, visualization, validation, software, methodology, formal analysis, conceptualization. **Weiyu Tao**: writing – original draft, validation, methodology. **Xinyu Qian**: writing – original draft, validation, methodology. **Qilong Liu**: writing – review and editing, resources. **Zhengyi Jin**: writing – review and editing, resources. **Ruina Kong**: writing – review and editing, supervision, conceptualization. **Jie Gao**: writing – review and editing, supervision, project administration, investigation, funding acquisition, conceptualization.

## Funding

This work was supported by grants from the National Natural Science Foundation of China (NSFC Project, Grant 82571999), the Natural Science Foundation of the Shanghai Higher Education Institutions of China (Grant 21ZR1478900), the Scientific research Project of Shanghai Municipal Health Commission (Grant 202340061), the Basic Medical Research Special Surface Cultivation Project of the First Affiliated Hospital of Naval Medical University (Grant 2023PY40), and the Training Program for Young Rheumatologists in Pujiang (Grant SPROG2406).

## Ethics Statement

All experimental protocols involving animals were reviewed and approved by the Animal Care and Use Committee of Shanghai Changhai Hospital (Approval Number CHEC2020‐105). Animal care and experimental procedures complied with relevant institutional guidelines and the National Institutes of Health Guide for the Care and Use of Laboratory Animals.

## Consent

Written informed consent was obtained from all individuals.

## Conflicts of Interest

The authors declare no conflicts of interest.

## Supporting Information

Additional supporting information can be found online in the Supporting Information section.

## Supporting information


**Supporting Information 1** Table S1: Primer used for RT‐PCR.

**Supporting Information 2** Additional file S2: Correlation analyses were performed between these four genes and the entire gene set. Heatmaps were generated to visualize the expression patterns of the top 50 positively correlated genes for each of the four genes.

**Supporting Information 3** Additional file S3: miRNA and transcription factors upstream of the predicted genes, from RegNetwork.

**Supporting Information 4** Additional file S4: Functional enrichment analysis of top100 marker genes from each cell. A KEGG pathway and B Reactome pathway.

## Data Availability

The public datasets were downloaded and analyzed in this study, which were available in the GEO data repository and the accession numbers were listed below: GSE89408 and GSE43292.
