# Supplementary material for: Identification of Immune‐Related Lactylation Genes in Rheumatoid Arthritis With Atherosclerosis: A Comprehensive Analysis Using Bulk and Single‐Cell RNA Sequencing Data
Source: Mediators Inflamm. 2026 May 11;2026:9969894. doi: 10.1155/mi/9969894 (PMC13159083; doi:10.1155/mi/9969894)
Supplement: Supplementary file 1 — Supporting Information 1 Table S1: Primer used for RT‐PCR. [file MI-2026-9969894-s001.pdf]

Supplementary Table 1 Primer sequence of target gene

Organisms	Gene	Forward primer (5' -3')	Reverse primer (5' -3')
Mus musculus	SMARCC2	AGCTCTGCTTCCCAGCAAAT	TTGCTGCAGCTTTGCTCTTG
	CCNA2	GCTGCATCAGGAAGACCAAGA	CTTAAGAGGAGCAACCCGTCGAG
	NUP50	ACGAAGTTGAAGAGATGGGAAC	TTCCTCCAGAGCCACCAAATC
	GATAD2B	GGGTCACAGTGTCATCCGTT	ACGAACTTGACTGTGGTTGGT
