# Supplementary figures and images for: Identification of Immune‐Related Lactylation Genes in Rheumatoid Arthritis With Atherosclerosis: A Comprehensive Analysis Using Bulk and Single‐Cell RNA Sequencing Data
Source: Mediators Inflamm. 2026 May 11;2026:9969894. doi: 10.1155/mi/9969894 (PMC13159083; doi:10.1155/mi/9969894)

CCNA2

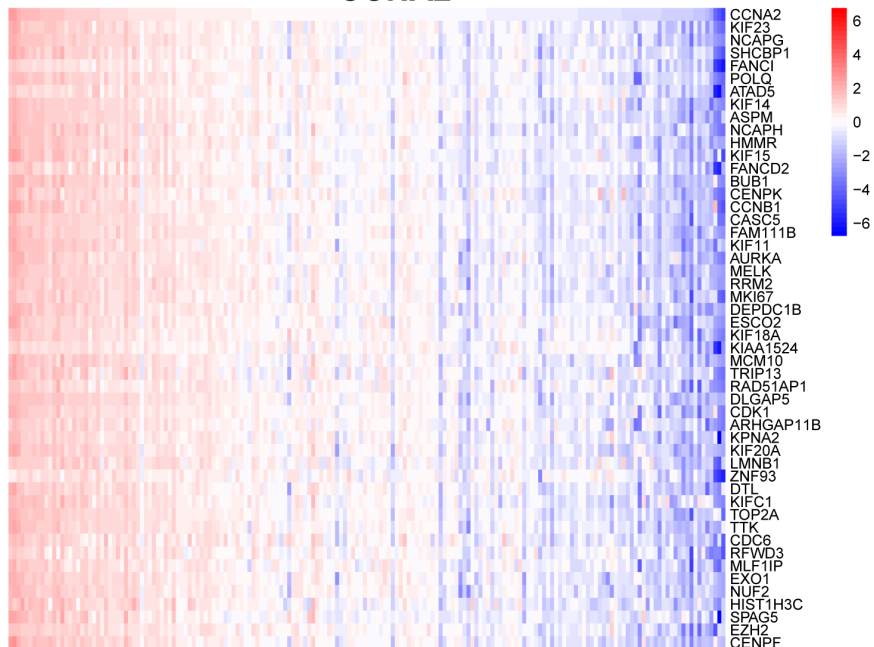

GATAD2B

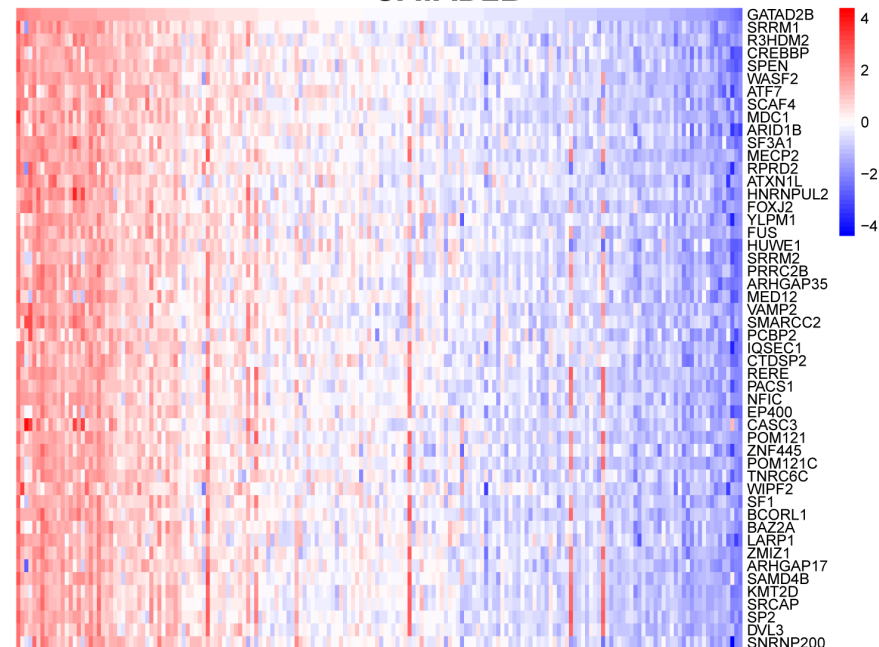

NUP50

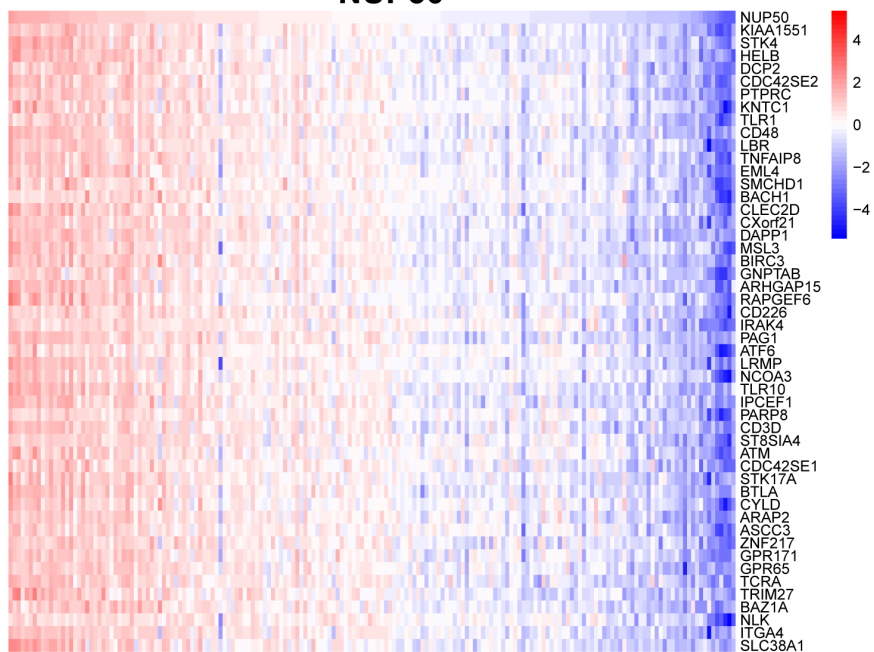

SMARCC2

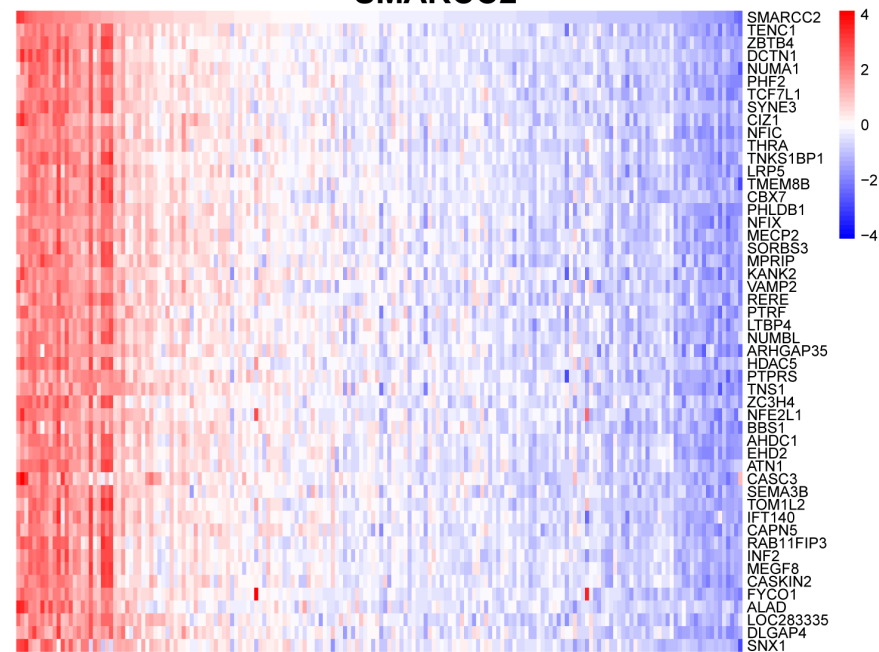

Supplement: Supplementary file 2 — Supporting Information 2 Additional file S2: Correlation analyses were performed between these four genes and the entire gene set. Heatmaps were generated to visualize the expression patterns of the top 50 positively correlated genes for each of the four genes. [file MI-2026-9969894-s002.pdf]

## Gene Regulatory Network

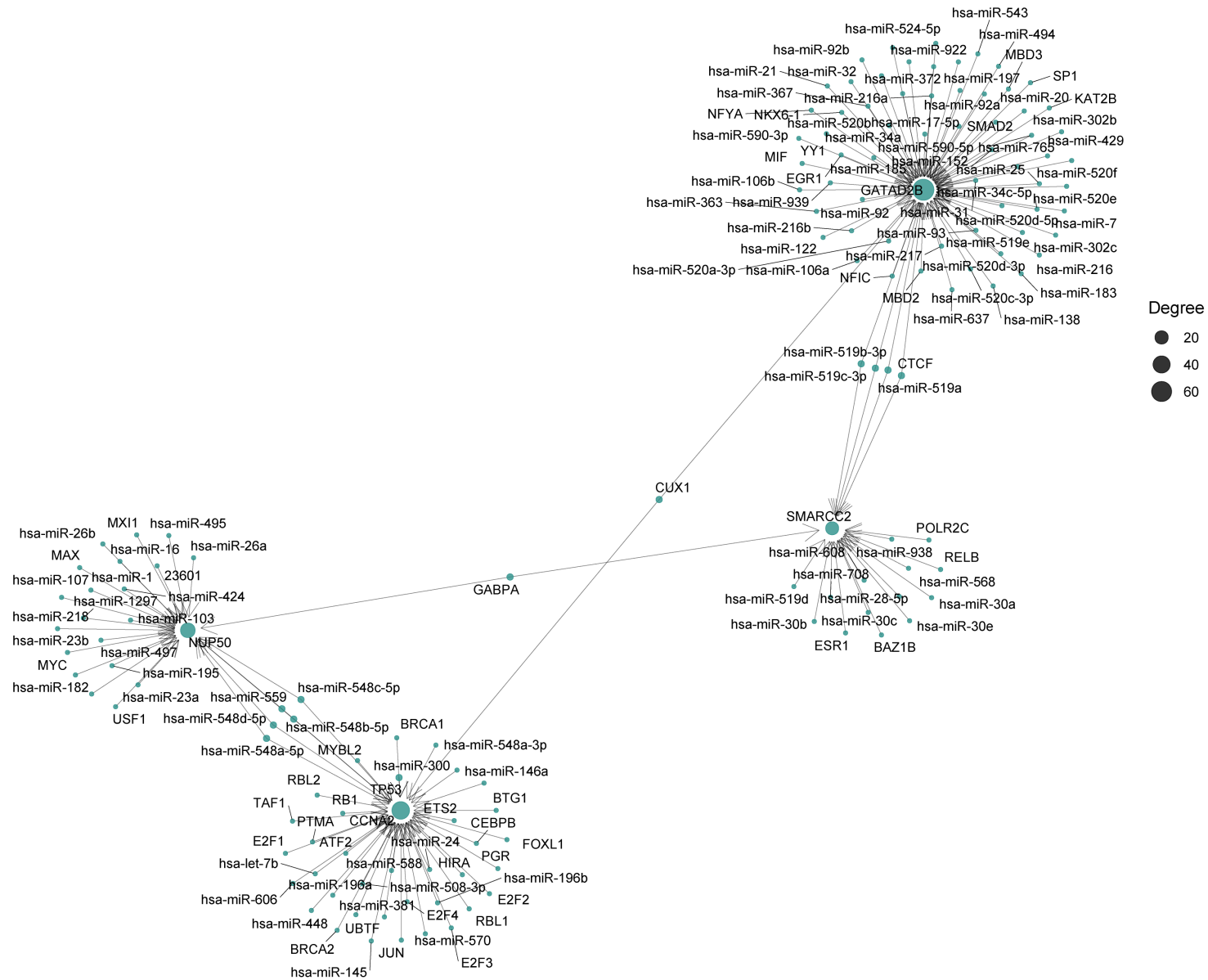

Supplement: Supplementary file 3 — Supporting Information 3 Additional file S3: miRNA and transcription factors upstream of the predicted genes, from RegNetwork. [file MI-2026-9969894-s003.pdf]

**A**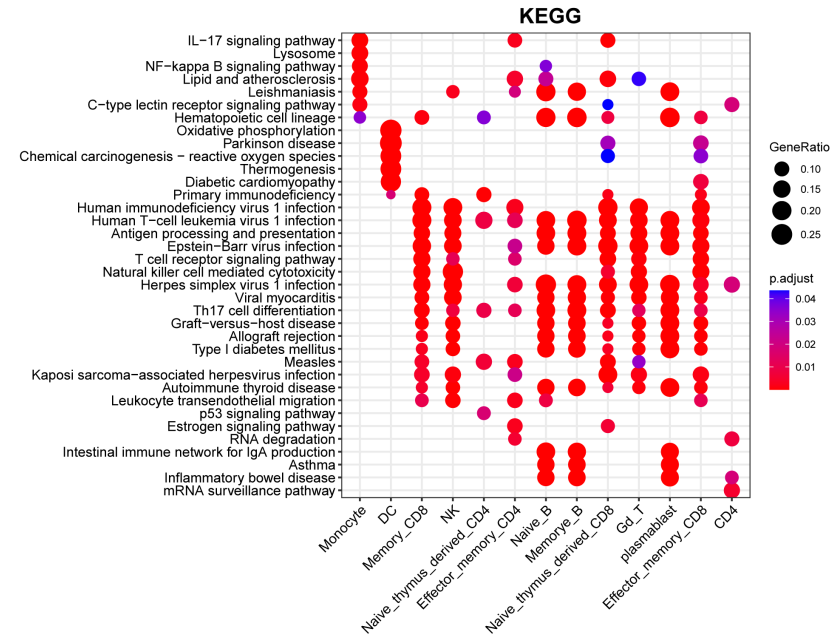**B**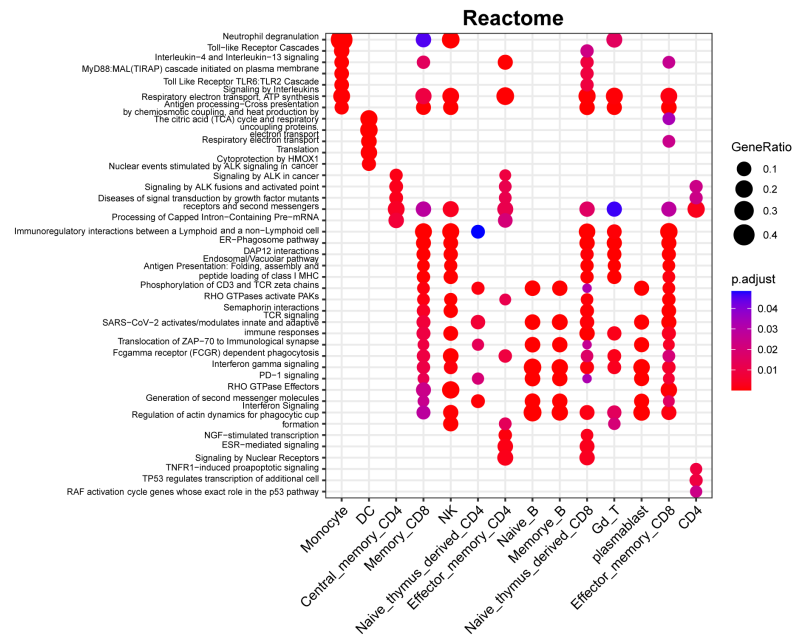

Supplement: Supplementary file 4 — Supporting Information 4 Additional file S4: Functional enrichment analysis of top100 marker genes from each cell. A KEGG pathway and B Reactome pathway. [file MI-2026-9969894-s004.pdf]
